# Measuring Behavior in the Home Cage: Study Design, Applications, Challenges, and Perspectives

**DOI:** 10.3389/fnbeh.2021.735387

**Published:** 2021-09-24

**Authors:** Fabrizio Grieco, Briana J. Bernstein, Barbara Biemans, Lior Bikovski, C. Joseph Burnett, Jesse D. Cushman, Elsbeth A. van Dam, Sydney A. Fry, Bar Richmond-Hacham, Judith R. Homberg, Martien J. H. Kas, Helmut W. Kessels, Bastijn Koopmans, Michael J. Krashes, Vaishnav Krishnan, Sreemathi Logan, Maarten Loos, Katharine E. McCann, Qendresa Parduzi, Chaim G. Pick, Thomas D. Prevot, Gernot Riedel, Lianne Robinson, Mina Sadighi, August B. Smit, William Sonntag, Reinko F. Roelofs, Ruud A.J. Tegelenbosch, Lucas P.J.J. Noldus

**Affiliations:** ^1^Noldus Information Technology BV, Wageningen, Netherlands; ^2^Neurobiology Laboratory, National Institute of Environmental Health Sciences, National Institutes of Health, Research Triangle Park, NC, United States; ^3^Roche Innovation Center Basel, Basel, Switzerland; ^4^Myers Neuro-Behavioral Core Facility, Sackler Faculty of Medicine, Tel Aviv University, Tel Aviv, Israel; ^5^School of Behavioral Sciences, Netanya Academic College, Netanya, Israel; ^6^Nash Family Department of Neuroscience, Icahn School of Medicine at Mount Sinai, New York, NY, United States; ^7^Department of Anatomy and Anthropology, Sackler School of Medicine, Tel Aviv University, Tel Aviv, Israel; ^8^Department of Cognitive Neuroscience, Donders Institute for Brain, Cognition and Behaviour, Radboud University Nijmegen Medical Centre, Nijmegen, Netherlands; ^9^Groningen Institute for Evolutionary Life Sciences, University of Groningen, Groningen, Netherlands; ^10^Swammerdam Institute for Life Sciences, University of Amsterdam, Amsterdam, Netherlands; ^11^Sylics (Synaptologics BV), Amsterdam, Netherlands; ^12^National Institute of Diabetes and Digestive and Kidney Diseases, National Institutes of Health, Bethesda, MD, United States; ^13^Laboratory of Epilepsy and Emotional Behavior, Baylor Comprehensive Epilepsy Center, Departments of Neurology, Neuroscience, and Psychiatry & Behavioral Sciences, Baylor College of Medicine, Houston, TX, United States; ^14^Department of Rehabilitation Sciences, College of Allied Health, University of Oklahoma Health Sciences Center, Oklahoma City, OK, United States; ^15^Sagol School of Neuroscience, Tel Aviv University, Tel Aviv, Israel; ^16^The Dr. Miriam and Sheldon G. Adelson Chair and Center for the Biology of Addictive Diseases, Tel Aviv University, Tel Aviv, Israel; ^17^Centre for Addiction and Mental Health and Department of Psychiatry, University of Toronto, Toronto, ON, Canada; ^18^Institute of Medical Sciences, University of Aberdeen, Aberdeen, United Kingdom; ^19^Department of Molecular and Cellular Neurobiology, Center for Neurogenomics and Cognitive Research, VU University Amsterdam, Amsterdam, Netherlands; ^20^Department of Biochemistry & Molecular Biology, Center for Geroscience, University of Oklahoma Health Sciences Center, Oklahoma City, OK, United States; ^21^Department of Biophysics, Donders Institute for Brain, Cognition and Behaviour, Radboud University, Nijmegen, Netherlands

**Keywords:** rodent behavior, neuroscience, home-cage, PhenoTyper, EthoVision XT, video-tracking

## Abstract

The reproducibility crisis (or replication crisis) in biomedical research is a particularly existential and under-addressed issue in the field of behavioral neuroscience, where, in spite of efforts to standardize testing and assay protocols, several known and unknown sources of confounding environmental factors add to variance. Human interference is a major contributor to variability both within and across laboratories, as well as novelty-induced anxiety. Attempts to reduce human interference and to measure more "natural" behaviors in subjects has led to the development of automated home-cage monitoring systems. These systems enable prolonged and longitudinal recordings, and provide large continuous measures of spontaneous behavior that can be analyzed across multiple time scales. In this review, a diverse team of neuroscientists and product developers share their experiences using such an automated monitoring system that combines Noldus PhenoTyper^®^ home-cages and the video-based tracking software, EthoVision^®^ XT, to extract digital biomarkers of motor, emotional, social and cognitive behavior. After presenting our working definition of a “home-cage”, we compare home-cage testing with more conventional out-of-cage tests (e.g., the open field) and outline the various advantages of the former, including opportunities for within-subject analyses and assessments of circadian and ultradian activity. Next, we address technical issues pertaining to the acquisition of behavioral data, such as the fine-tuning of the tracking software and the potential for integration with biotelemetry and optogenetics. Finally, we provide guidance on which behavioral measures to emphasize, how to filter, segment, and analyze behavior, and how to use analysis scripts. We summarize how the PhenoTyper has applications to study neuropharmacology as well as animal models of neurodegenerative and neuropsychiatric illness. Looking forward, we examine current challenges and the impact of new developments. Examples include the automated recognition of specific behaviors, unambiguous tracking of individuals in a social context, the development of more animal-centered measures of behavior and ways of dealing with large datasets. Together, we advocate that by embracing standardized home-cage monitoring platforms like the PhenoTyper, we are poised to directly assess issues pertaining to reproducibility, and more importantly, measure features of rodent behavior under more ethologically relevant scenarios.

## Introduction

Reproducibility of research, i.e., the ability of researchers to duplicate the results of a prior study (Goodman et al., 2016) is a growing concern in preclinical behavioral sciences (Steckler et al., 2015; Loken and Gelman, 2017; Voikar, 2020). An array of causative factors have been identified, including methodological discrepancies, variations in analysis and reporting structures, and differences in the conclusions between replicates of a study (Goodman et al., 2016). As in other fields of biomedical research, preclinical studies must be reproducible, particularly when dealing with the behavior of laboratory animals, which is highly sensitive to environmental factors (Sousa et al., 2006). Although numerous tests of behaviors in many domains are readily available (Hånell and Marklund, 2014), lab-specific protocols prevail, even for simple tests like the open field (Wahlsten, 2001). The many limitations of conventional battery-based assays have been widely acknowledged (Gerlai, 2002; Wahlsten et al., 2003; Tecott and Nestler, 2004; Spruijt and De Visser, 2006; Kalueff et al., 2007; Kas et al., 2008; Mandillo et al., 2008; Spruijt et al., 2014; Freudenberg et al., 2018). For example, behaviors related to anxiety are species-specific and apparatus-specific (O’Leary et al., 2013), which further increase variability and dampen relevance of animal models. These “standard” assays/tests are short-lasting and depend on the subject’s activity levels and the subject’s responsiveness to the novel environment. Besides, standard tests may be good to investigate to what extent an intervention causes a biologically relevant effect, but less suitable to assign a brain function to those behavioral changes. For example, the time spent in the center in the open field may reliably reflect the immediate effect of a treatment with anxiolytic drugs (that is, behavior as an indicator in a bioassay), but its interpretation in terms of “anxiety” may be oversimplified (Spruijt et al., 2014).

In recent years, home-cage monitoring systems (HCMS) have been developed as an attempt to complement standard behavioral tests. First, such systems allow prolonged unbiased observations of spontaneous behavior. If the levels of activity normally displayed by the subjects under baseline conditions are known, then interpretation of data obtained in tests for exploration or anxiety is facilitated (Tang et al., 2002). Second, studying subjects in their familiar environment results in more naturalistic observations (Olsson et al., 2003; Wolfer et al., 2004) and reduces novelty-related interferences, which is relevant especially when conducting batteries of tests (Kas and van Ree, 2004) and when studying stress or anxiety-related behaviors (Kyriakou et al., 2018). HCMS should be flexible enough to integrate multimodal data acquisition (video, physiological data, etc.) and implement controlled and standardized environmental perturbations (Würbel and Garner, 2007).

HCMS rely on different technologies to detect and quantify the behavior of animals. Past reviews described some benefits of HCMS with different settings (Spruijt and De Visser, 2006; Robinson and Riedel, 2014; Richardson, 2015; Voikar and Gaburro, 2020). In this review, we share our experiences using the PhenoTyper, developed by Noldus Information Technology, combined with the video-based tracking software EthoVision XT. Unlike standard home-cages found in the vivarium, the PhenoTyper is optimized for comprehensive recording of behavior of rodents with an overhead camera combined with the use of software-controlled stimuli for a variety of behavioral tests and automated use of hardware like food dispensers (see "What Makes a Cage a Home Cage" and "Data Acquisition" Section). The aim of this review is to highlight the techniques for measuring the behavior in HCMS such as the PhenoTyper. We demonstrate how such systems can complement standard tests to produce digital biomarkers, defined here as quantifiable, physiological and behavioral data that are measured and evaluated as indicators of biological processes, which contribute to the quality of preclinical research and improve our understanding of behavior. Because none of the test apparatuses and methods currently available, including HCMS, can address all the issues related to behavioral research in the laboratory, understanding the advantages and disadvantages of different methods is crucial when deciding on the proper testing applications and analyzing the results.

## Benefits and Limitations of A Home-Cage Monitoring System

### What Makes a Cage a Home Cage

In designing appropriate housing conditions for prolonged monitoring, which we define, somewhat arbitrarily, as any observation covering at least one complete light or dark phase, perhaps the most important consideration is to minimize observer effects, defined as the disturbance of an observed system by the act of observation itself. In most articles included in this review, continuous monitoring ranges between 3 days (e.g., Kas et al., 2008; de Mooij-van Malsen et al., 2009; Cao et al., 2018) to 4 weeks (de Visser et al., 2007). Additionally, to be in line with animal welfare regulations^1^, the design of HCMS should crucially promote species-specific natural behavior. Whenever possible, such systems should be furnished with familiar bedding (Blom et al., 1996), chow and drinking water identical to those of vivarium conditions (Jankovic et al., 2019; Bass et al., 2020). Additionally, environmental enrichment by means of additional substrates and objects allows to expand the range of behaviors that the animals can express (Wolfer et al., 2004; Baumans, 2005). Providing adequate cage ventilation is critical, and bedding may need to be adapted to maximize contrast for variety of mouse/rat coat colors when subjects are video-tracked. Ideal cage sizes may vary depending on the objectives of the experiment. For instance, prolonged multiday recordings, spaced out over months of the subject’s lifespan (Dowse et al., 2010; Ahloy-Dallaire et al., 2019) may benefit from larger cages as they permit less frequent cage cleaning. There have been several recent studies demonstrating the utility of non-commercial monitoring systems applied directly to conventional rack-mounted cages. Most of these creative solutions measure activity through video analysis (Singh et al., 2019) or under-cage capacitive plates (Pernold et al., 2019), in contrast to traditional beam-break grids (Angelakos et al., 2019). One recent study demonstrated the utility of a simple fixture to measure feeding and body weight, which can substantially change throughout the day (Ahloy-Dallaire et al., 2019). Finally, simultaneous tethered recordings of EEG/ECG or those that incorporate optogenetic techniques benefit from a cage that is both wide and tall to avoid tether tangling.

**Figure 1:**
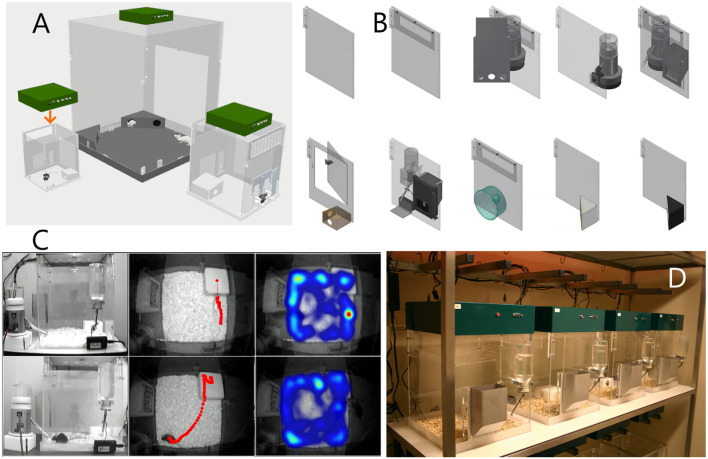
The four pillars of Noldus PhenoTyper as a home cage monitoring system. **(A)** Different cage sizes can be combined with the same device (the Top Unit) that functions as the interface between the cage and the video-tracking system. Left, 30 × 30 cm cage for single mouse; middle, 90 × 90 cm for rat social interaction; might, 45 × 45 cm for single rat or mouse social interaction. **(B)** Cages can be made of different functional components, easily assembled, disassembled and cleaned. **(C)** Control of stimuli and recording of behavior and analysis is performed by the EthoVision XT software. Left: external view of two cages during a conditioning experiment where the mouse must sit on top of the shelter in order to receive a reward. Cages are provided with a pellet dispenser for the rewards and a lickometer to additionally measure drinking behavior. Middle: view from the Top Unit with tracks. In the cage at the top, the dot on the shelter indicates that the mouse is inside the shelter; the time spent in the shelter is also measured. In the other cage, the mouse has just received a reward after it was detected on the top of the shelter. Right: example of locomotor/exploration behavior visualized as heatmaps. **(D)** Cages can be placed in standard racks and tests are performed simultaneously, with up to 16 cages per EthoVision XT workstation. The subjects are usually released and taken by lifting the feeding tray.

Under conditions of social isolation, drifts in behavioral features may be seen. This may be related to infradian rhythms or the cumulative effects of social isolation itself. In one study employing PhenoTyper cages studying socially-isolated C57BL/6J mice during an 8 day recording period, total daily distances gradually declined with similar gradual increases in total daily sleep (defined behaviorally) as well as feeding and licking times (Bass et al., 2020). Providing greater environmental enrichment may ameliorate such phenotypic drifts. These include running wheels (de Visser et al., 2005, 2007) which provide an avenue to clarify whether changes in measures of horizontal displacement (hypo- vs. hyperactivity) extend to measures of voluntary exercise. Since wheel access may be sufficient to ameliorate several aspects of neuropsychiatric symptomatology in rodent models (Guo et al., 2020), a single day of wheel access can be incorporated into a home-cage based battery of behavioral testing (Bass et al., 2020) using detachable running wheels for the PhenoTyper. To prevent the effects that may come about from social isolation, several recent reports demonstrate the feasibility of studying rodents in pairs or larger groups using RFID-based individual identification, which can be applied to home-cages (Alexandrov et al., 2015; de Chaumont et al., 2019; Peleh et al., 2019, 2020). Without RFID chips, or fluorescent markers (Shemesh et al., 2013), infrared video recordings are somewhat limited in their ability to distinguish between individual subjects within a group (Bass et al., 2020), and therefore report on measures that pertain to the group as a whole (e.g., total distances of the pair, mean proximity between subjects of the pair). Thus, a suitable home cage for HCMS studies is one that provides food, water, shelter and bedding, and optionally other enrichment objects. The PhenoTyper meets these specifications, and was uniquely designed *de novo* to capture subjects with an overhead camera, and allow variation in cage size and wall configurations (Figure 1). It is not necessarily meant as the permanent residence of the animal, as it can function as experiment cage for multi-day tests. It still requires habituation after the subjects are transferred from the vivarium. The habituation phase depends on several factors including the subjects’ strain (Loos et al., 2014; Bass et al., 2020; see “Reducing Human Interference and Controlling for the Effects of Habituation” Section).

### A Comparison With “Standard” Tests

“Standard” tests, like the open field or the elevated plus maze tests, capture brief snapshots of behavior at pre-determined time points, whereas HCMS track behavior for longer periods. This allows for continuous and longitudinal monitoring of a subject’s behavior using automated recording of movement, interaction with stimulus and response devices, and body posture changes, capturing effects that may not be observable with classical short tests. One of the most important benefits of HCMS is the ability to assess behavior continuously, especially in studies where novel manipulations (e.g., pharmacological or genetic) are explored and there is no information available regarding behavioral changes over time. In some rodent models of various disorders, abnormal behavior tends to be subtle and difficult to capture in short testing regimen, or during health assessment where mice may hide signs of poor health from the human handler that they may consider as a potential predator (Mayer, 2007). Additionally, the novelty aspects of standard tests (such as square or circular open fields) may either exaggerate or attenuate the genetic or pharmacological manipulations studied. Another benefit of home-cage testing is the ability to assess and track behaviors in a relatively stress-free environment that allows activity at the subject’s pace. This can promote faster learning of tasks and may highlight differences of behaviors that are absent when using short-sampling standard methods (Remmelink et al., 2014; Remmelink et al., 2016b).

On the other hand, one advantage in using standard tests, as opposed to HCMS, is the ability of using extreme motivational conditions at certain time-points (e.g., foot-shock), whereas HCMS are designed to assess behavior in a relatively stress-free environment over a long period of time. Therefore, while HCMS can offer certain stressors to assess anxiety behaviors (e.g., spot of light; Prevot et al., 2019b; Bass et al., 2020), they are currently deficient in their ability to provide strong aversive cues, such as the footshock or the air puff given based on the subject’s behavior in the home cage.

One of the most critical factors to be considered when designing a test battery is the stress impact that each task has on the outcome of the following test when using the same subject (McIlwain et al., 2001) or the number of times that the subject is exposed to the test (Paylor et al., 2006). Similarly, using an aversive stimulus in a home cage environment will have an effect on the behavior of the subject in the home cage itself, that is, the environment that is supposed to be familiar and safe. This effect may occur at later stages, for example during prolonged recordings.

### Controlling for Environmental Variation

**Figure 2:**
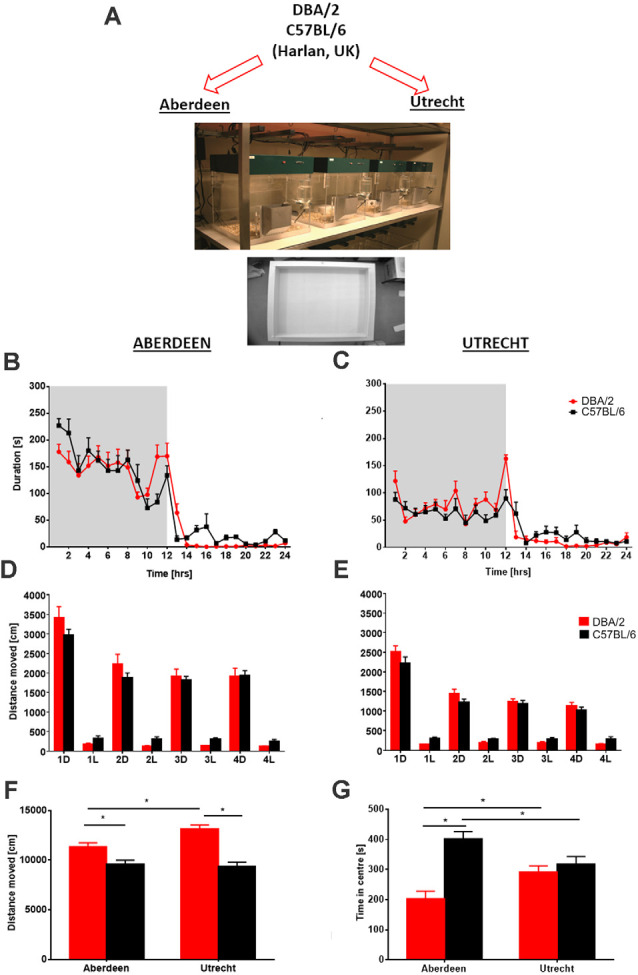
Between laboratory analysis of ambulatory activity and anxiety-related behavior in two different mouse strains. **(A)** Following delivery of mice to the two behavioral facilities in Aberdeen and Utrecht, identical experiments were conducted using the home cage observation system PhenoTyper and the open field. Circadian activity (hourly bins) expressed as time spent in the open area of the PhenoTyper during a 24-h period (shaded area = dark phase of testing) revealed that activity of both mouse strains DBA/2 and C57BL/6 in Aberdeen **(B)** and Utrecht **(C)** laboratories was increased during periods of darkness and declined during the light phase. Despite overall higher ambulatory activity in the Aberdeen mice, similar activity peaks at the beginning and end of the dark phase were obtained with both strains in both laboratories. Analysis of distance moved across four consecutive recording days averaged for 12-h time bins, dark (D) and light (L) phases of activity revealed no overall significant differences between strains in both **(D)** Aberdeen and **(E)** Utrecht, although similar trends were observed across laboratories with DBA/2 mice being more active during the dark phases and C57BL/6 more active during the light phases. Following completion of PhenoTyper testing analysis of activity (distance moved) **(F)** and anxiety-related (time spent in the center) in the open field **(G)** revealed activity differences between the two strains that were comparable across both laboratories, with DBA/2 mice displaying higher levels of activity than C57BL/6 mice. However, a difference in anxiety-related behavior between the two strains was only observed in Aberdeen with DBA/2 displaying heightened levels of anxiety-like behavior (i.e., less time spent in the center) compared to C57BL/6. Furthermore, some strain differences were observed between laboratories with C57BL/6 mice being less anxious in Aberdeen compared to Utrecht with the opposite observed for DBA/2. Data are presented as means + SEM. Asterisks denote *p* < 0.05, *t*-test. The figure is adapted from Robinson et al. (2018).

The phenotype of animals, including humans, is the product of the interaction between their genotype and environment (Würbel, 2002). Environmental variables include housing and experimental conditions such as room temperature, humidity, cage type (open, filtertop, individually ventilated), cage cleaning, position of cage on shelves of the rack in the housing room, smells, experimental design, handling, day of experiment, lighting and time of the day and order of testing, noise in the animal facility (Homberg et al., 2010; Bohlen et al., 2014; Shan et al., 2014; Robinson et al., 2018), which all can influence reproducibility. For example, the time of day in which an animal is tested can have huge impacts on standard behavioral assays and is a common protocol difference between labs (Bodden et al., 2019). Behavioral phenotyping using an automated home-cage environment has distinct advantages over conventional behavioral assays and can be valuable in standardizing testing paradigms (Robinson and Riedel, 2014; Robinson et al., 2018; Arroyo-Araujo et al., 2019) because they limit many of the experimenter interactions and use uniform protocols. For example, investigation of ambulatory activity of inbred mouse strains (DBA/2 and C57BL/6) across two laboratories (Aberdeen and Utrecht) utilizing the PhenoTyper with standardization of housing and testing conditions produced consistent strain differences in the two laboratories. Home-cage observation facilitated reproducibility of activity-related but not anxiety-related phenotypes in the open-field test, with PhenoTypers eliminating environmental factors that influenced reliability (Robinson and Riedel, 2014; Robinson et al., 2018; Figure 2). Finally, there was a high degree of reliability when different cohorts of young animals were tested in consecutive years using a discrimination test (Remmelink et al., 2016b). These findings indicate that standardization of behavioral tests within and between laboratories is possible and necessary. Nevertheless, a potential caveat to this concept is that excessive standardization may increase repeatability of results within and between labs, which has already been shown, but at the same time reduce the external validity, that is, the extent to which those results can be generalized to a wider range of experimental conditions, strains etc. (Würbel, 2000; Voelkl et al., 2020).

### Reducing Human Interference and Controlling for the Effects of Habituation

Researchers often target physiology and behaviors that are sensitive to external sources of stress; this stress can adversely impact the results of a study, for example by increasing the heart rate, respiratory rate, and altering activity levels and exploratory behavior. Furthermore, it can complicate replication of a study and introduce seemingly random sources of variation into datasets. To combat this, HCMS allow to target two interventions: handling and environmental habituation (Deacon, 2006).

Handling has a major impact on the animal’s anxiety (Hurst and West, 2010; Gouveia and Hurst, 2017, 2019). In general, human/experimenter intervention is a critical factor influencing reproducibility (Chesler et al., 2002a,b; Kas and van Ree, 2004; Sorge et al., 2014). For instance, a recent study found that exposure of rodents to a male experimenter causes high stress and pain inhibition (Sorge et al., 2014). Further, a study on mice tested within three different laboratory settings found variation in mouse behavior due to an experimenter in one lab who was allergic to mice and wearing a respirator while conducting the test (Crabbe et al., 1999). Another study reported that rats were capable of recognizing a familiar experimenter from unfamiliar people with significant impact on anxiety and exploratory behavior (Morlock et al., 1971; van Driel and Talling, 2005).

Handling a rodent by the tail is stress-provoking (e.g., Clarkson et al., 2018), but gradual and consistent handling will reduce rodents’ stress upon experimenter handling. Handling requirements also must be tailored to *in vivo* procedures, including intraperitoneal or subcutaneous injections, or subject attachment to external equipment including fiber-optic cables, microfluidic pumps for localized fluid delivery, and *in vivo* microscopy apparatuses. Pre-experimental handling can produce marked effects on many behaviors in mice and rats, including anxiety- or stress-related behaviors (Levine et al., 1967; Wakshlak and Weinstock, 1990) and memory tasks (Costa et al., 2012). This may even vary with strain-based differences in physiological perturbations (Van Bogaert et al., 2006), highlighting the need to control this variable as much as possible.

Environmental habituation presents a more consistent and time-consuming stressor to overcome. Many research facilities do not maintain animal husbandry in the same rooms as experimental testing rooms, meaning researchers must move test subjects out of their colony room into a waiting area for experiments. This transfer provides a new set of cues: visual, olfactory, and auditory stimuli, which may exaggerate or attenuate phenotypic differences brought on by genetic or pharmacological manipulations. Many protocols add at least 1 h of habituation time into procedures to allow animals to adjust to their new surroundings before a behavioral experiment begins.

A prime example of behaviors sensitive to habituation is the rich suite of social behaviors expressed by laboratory rodents. Social behaviors are highly context-dependent, relying on factors including territorial ownership (Collias, 1944), social partner (Yang et al., 2017), prior experience (Archer, 1976) and even reproductive status (Wolff, 1985; De Almeida et al., 2014) for expression of pro-adaptive social behaviors. Exploration of the biological underpinnings of social behavior requires careful control of environmental factors to optimize behavioral manipulations. Male rodent aggressive behavior, for example, is expressed most consistently after establishing territorial ownership with extensive scent-marking (Collias, 1944) and is influenced by previous encounters (Dugatkin, 1997; Hsu et al., 2006). Likewise, more recently-discovered aspects of female aggression require habituation to a cohabitation partner, which is deemed vital to provoke aggression towards same-sex intruders (Newman et al., 2019). Minimizing introduction of conflating variables, including changes in home-cage environment, are vital to eliciting optimal social phenotypes for investigation. This was demonstrated recently in studies investigating real-time behavioral choice between social interaction with a novel conspecific and food intake in different need states (Burnett et al., 2019).

### Prolonged Observation of Behavior

Continuous monitoring (i.e., non-stop observations lasting for several up to 28 days) and longitudinal studies (i.e., repeated recording during aging of the subject, for example at 3, 6, 12 and 24 months of age) proved to be necessary to gain insights in behavioral readouts. For example, prolonged monitoring approaches help to uncover the interplay of genetic factors and time, like in a study of locomotor activity in four genetic mouse models for autism: Shank3^−/−^, Cntnap2^−/−^, Frm1^−/−^, and Pcdh10^+/−^. While previous studies report hyperactivity (e.g., Peier et al., 2000; Peñagarikano et al., 2011) or hypoactivity (Brunner et al., 2015; Mei et al., 2016) or no change (Peça et al., 2011) in acute testing situations like open field, multiple-week home-cage monitoring revealed a consistent hypoactivity in the dark phase in all four strains compared with their wild-type littermates (Angelakos et al., 2019). These findings align with abnormalities in rest and activity rhythms in Autism Spectrum Disorder patients (Höglund Carlsson et al., 2013; Posserud et al., 2018), and underscores the importance of home-cage testing to assess the translational values of preclinical models.

While some manipulations are stable throughout the light/dark cycle, other manipulations may affect behaviors during different phases (e.g., higher activity during one phase, not the other), or disrupt the cycle itself (e.g., subjects are hyperactive during the light phase; Jankovic et al., 2019). Longitudinal phenotyping can investigate behavioral changes in a circadian-dependent manner. Namdar et al. (2020) showed that mTBI affects mice activity. They measured daily activity by means of the home-cage running wheel, and showed that activity was lower during the active time period (i.e., during the dark cycle) and higher during the resting time period (i.e., during the light cycle) compared with control subjects. This reveals the difficulty mTBI mice have in maintaining their sleep cycle. A study in mouse models of Huntington’s disease (HD) substantiated the importance of longitudinal assessment of behavior. Repeated weekly measurements conducted until the age of 13 weeks unraveled previously unreported aberrant behaviors by showing different levels of activity during the dark and light phases. Combining a variety of behavioral features over time led to a much earlier classification of diseased mice than was previously possible through single behaviors (Steele et al., 2007).

### Limitations of HCMS

While the empirical studies reported in the following sections show that HCMS are a valuable addition to the toolkit for behavioral analysis, one should also consider their limitations, some of which are of very general nature and therefore shared with other methods. First, the design of the cage may pose constraints on the behavior that animals can express. More naturalistic test environments like the visible burrow system (Blanchard and Blanchard, 1989; Blanchard et al., 2001), create the context for naturally-occurring behavior, including interaction between multiple animals, adding translational value to the models. It is in principle possible to create visible burrows in a large PhenoTyper, by covering the inner chambers with infrared-translucent material (the issues pertaining monitoring of social behavior are discussed in Section “Social Behavior in the Home Cage”). Second, automated systems often output pre-defined behavioral readouts like velocity. Those variables are selected by humans, which may introduce biases in the findings (Golani, 2012; Pellis and Pellis, 2015). A way out of the problem is to segment the flow of behavior into blocks based on geometry or statistical properties of postures and movements. More animal-centered measures of behavior are further discussed in Section “Today and the Future”.

Monitoring for prolonged time usually requires observing behavior after the test has taken place. Limitations may occur when the observer relies on the offline video record, which may not show all behaviors because, for example, the subject was not entirely visible in the camera image. This can be ameliorated by adding cameras which provide a side view of the subject). Furthermore, some tasks like spatial memory tasks (Vorhees and Williams, 2014) may be difficult to implement when the task requires large spaces, or when the test needs to be spatially separated from the home-cage, however a tunnel connecting the home-cage to the test chamber may be a solution (Section “Measuring Learning and Cognitive Functions”).

## Data Acquisition

The PhenoTyper and other home-cage monitoring platforms offer unique insights into the structure of spontaneous behavior in rodent models (details on the applications are in Section “Measuring and Analyzing Behavior”). Fundamentally, such platforms should be capable of measuring dynamic changes in the subject’s horizontal displacement. Top-view video tracking on a distance-calibrated home-cage arena, as applied by the PhenoTyper in concert with EthoVision XT, provides distances moved per hour/minute/day. Coarse measurements of distances (e.g., cm/h) are sufficient to define rudimentary circadian variables (Loos et al., 2014). Wheel-running assessments within the PhenoTyper can distinguish between hyperactivity and changes in the motivation to voluntarily exercise. Using combinations of top-view video recording, add-on devices (shelter, pellet dispenser, etc.), auditory and visible stimuli (noise, lights) and advanced tracking system, the HCMS allows investigation of various behavioral domains. Whereas the interpretation of the readouts is the focus of Section “Measuring and Analyzing Behavior”, the present Section deals with technical issues related to the design of experiments with home-cage systems.

### Challenges for 24/7 Home-Cage Video Tracking

Of the many sensors available for behavioral monitoring, video imaging allows for the capture of most of the relevant information, which makes video tracking a popular technique. A home cage environment poses additional challenges to a video-based tracking system. We briefly discuss the most relevant ones.

#### Lighting

To allow continuous recordings in both dark and light periods, video tracking should be independent of room illumination. This can be achieved using an infrared-sensitive camera with an IR-pass filter, which blocks visible light, combined with constant illumination by infrared LED arrays placed above the tracking area. When using visible light, illumination should be even in order not to affect place preference, as rodents tend to spend more time in darker areas.

#### Background Noise

**Figure 3:**
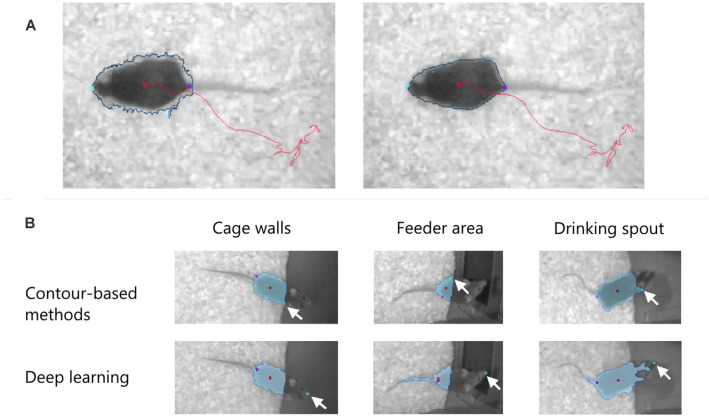
**(A)** Effect of pixel filtering to remove indentations in the subject contour (blue line) and make it less dependent on the spatial variation of the background. Left: before filtering. Right: after filtering. The nose, the tail-base, and the center of the body are shown with color dots. **(B)** Detection of the nose point in EthoVision XT 16 in three “difficult” cases where the mouse moves over dark surfaces in a PhenoTyper cage. Two methods are used to find the nose: contour-based and deep learning. The arrows indicate the position of the detected nose. In all cases, the deep learning method correctly finds the nose independent of the detected blob (in light blue).

Bedding and nesting material provided in the home cage create a grainy background. The software should not only remove this noise to increase the contrast of the subject to be tracked but also compensate for the temporal changes in the background, for example when a mouse makes a nest and displaces the bedding. The software should also smoothen the subject contour, removing indentations especially when one wants to quantify the mobility of the body (Figure 3A).

#### Track Smoothing

The uneven background adds noise to the tracked position of the animal. In EthoVision XT, smoothing algorithms based on locally-weighted regression (LOWESS; Hen et al., 2004) can be activated both during tracking and in the analysis phase. Track smoothing is particularly important in conditioning tasks, for example when the subject entering a target zone is supposed to trigger a stimulus. If random noise causes false positioning of the animal in the target zone, the trial protocol will be invalid.

#### Body Point Detection

When measuring exploratory or social behaviors, correct detection of specific body points like the nose is essential. In a recent development, trained deep neural networks find the animal’s nose more accurately than methods based on the contour of the detected blob. Figure 3B compares the performance of the two methods in EthoVision XT 16 when finding the nose of a mouse over a difficult background.

#### Shelters and the Like

The system should quantify the time that the animal is not detected as it sits inside the shelter. In EthoVision XT the shelter is defined as “hidden zone” (e.g., Maroteaux et al., 2012). By using shelters made in infrared-translucent material, one can also follow the subjects when they are inside the shelters (Jankovic et al., 2019; Bass et al., 2020). This simple solution does not, however, allow to distinguish between instances when the subject is in the shelter and when it sits on top of it. A second camera placed at one side of the cage or a depth camera may help solve that issue.

#### Multi-animal Tracking

Researchers advocate that animals should be studied in group-housed conditions (Peters et al., 2015), although that has to be considered carefully to avoid social stress and aggression (Kappel et al., 2017). The use of multiple animals in the same test chamber poses the problem of identifying individual animals. This pertains to all apparatuses, not only HCMS, and is being tackled in different ways, e.g., using RFID sensing for individual recognition (Bains et al., 2016), by combining camera tracking with RFID sensing (de Chaumont et al., 2019), or by video-tracking individually-marked subjects (Peters et al., 2017).

### Expanding the Home Cage: an Example With EEG Recording

The recordings of sleep-wake rhythms have always been performed in modified home cages. Exact determination of sleep staging, however, relies on the recording of synchronous, primarily dendritic activity from large populations of neurons originating in cortical columns by means of electroencephalography (EEG; see Swartz and Goldensohn, 1998 for a review). In rodents, EEG is an invasive procedure, with at least two subdural or intraparenchymal electrodes, typically connected by a tether to an amplifier and oscilloscope or polygraph (Wetzel and Matthies, 1986). The tethering may interfere with the video-base observation of the subject, however software like EthoVision XT can remove the effect of the tether by filtering the contour of the detected subject. Sleep staging can be conducted based on the EEG traces and electromyography (EMG), and may be combined with information about movement or location of the subject to provide a richer understanding of the behavioral correlates of EEG oscillatory activity.

Can sleep be determined by video analysis alone? There is no short answer to this question. Multiple laboratories have recorded the home-cage activity of rats or mice through video-analysis and developed analysis algorithms to distinguish between wake and sleep stages. They came to the conclusion that extended periods of immobility longer than 40 s seemingly reflect sleep in mice (Pack et al., 2007; Singh et al., 2019), but a more refined characterization of and transitions between states of immobility [which may reflect slow wave sleep, rapid eye movement (REM) sleep or even quiet wakefulness] are impossible without simultaneous EEG (Fisher et al., 2012; Brown et al., 2017).

Avoiding the use of tethered EEG equipment was a pre-requisite for the successful set-up of sleep recordings in the PhenoTyper. A number of biosensors for data logging have been developed, for example the Neurologger (Vyssotski et al., 2006; Jyoti et al., 2010, 2015; Platt et al., 2011; Goonawardena et al., 2015) and the NAT-1 (Crouch et al., 2018, 2019; Crispin-Bailey et al., 2019). Both are wearable ultra-miniature devices of light weight (<3 g), record four channels at ≥200 Hz sampling frequency, have on board memory of more than 512 MB, come with infrared (IR) sensors for event-synchronization, carry a 2D or 3D accelerometer making implantation of electromyographic electrodes obsolete, and record for up to 7 days. These head-mounted devices are easily carried by a mouse without alterations to their circadian activity (Jyoti et al., 2010) and without interference with the video signal. Other widely used EEG recording equipment avoiding tethers developed by Data Science International utilizes a single channel recording through a battery powered transponder implanted under the skin of the subject (Weiergräber et al., 2005). The lack of on-board memory requires the continuous download from the transmitter to a receiver plate typically placed under the behavioral recording equipment. These devices have been applied in multiple experimental settings in transgenic mice and in drug studies. Data showed that despite normal circadian activity recorded *via* EthoVision in the PhenoTyper, triple transgenic Alzheimer mice displayed an age-related slowing of the EEG and an increase in short episodes (<40 s) of non-REM sleep (Platt et al., 2011; Jyoti et al., 2015). This has several implications: (i) scientifically, the model mimics the human patient and constitutes a biomarker for disease state; (ii) technically, it questions the suitability of purely video-based sleep scoring highlighted above and suggests that considerable amounts of sleep go undetected if a 40 s threshold is applied; and (iii) collectively, the outcome provides compelling evidence that the overall activity profile derived from video observations like those obtained with EthoVision XT and detailed sleep patterns are not congruent but complementary and require independent recording.

### Controlling Stimulus Presentation in the Home Cage

The study of natural behaviors in a home cage environment provides a tremendous opportunity to improve our understanding of behavior in general, and discriminate mouse mutants, pharmacological challenges and other interventions. This ethological view on mouse behavior may however not satisfy researchers interested in translational research. More specifically, it is not immediately evident how changes in specific mouse behaviors translate to clinically relevant behavioral changes in humans. Hence, over recent years examples of translational behaviorist approaches have been published that use specific stimuli in order to evoke responses that are considered translationally relevant. Stimuli typically employed in the PhenoTyper home-cage include LED lights provided in the top unit or custom developed peripherals (e.g., shelter lights), pure tones that can be strobed or timed to a specific behavior, as well as food rewards that can be dispensed using a dispenser coupled to the cage (Maroteaux et al., 2012; Aarts et al., 2015; Remmelink et al., 2016a). The EthoVision trial and hardware control functions can be used to detect the location of the animal in real-time and trigger stimuli to occur, and thereby reinforcing certain behaviors while suppressing others. In the section Analysis of Behavior, several examples are provided that used real-time hardware control to measure anxiety-related behavior as well as aspects of associative and instrumental learning.

### Recording Vocalizations in the Home Cage

Rodents display a wide range of ultrasonic vocalizations (USVs) in response to various situations, especially during social interactions (Holy and Guo, 2005; Portfors, 2007; Takahashi et al., 2010). USVs are an important component of a behavioral phenotype (Scattoni et al., 2009; Simola and Granon, 2019; Hobson et al., 2020) and have been successfully used to investigate, among others, communicative deficits in Autism Spectrum Disorder models (Ey et al., 2012; Wöhr, 2014; Ferhat et al., 2016) and age-related degenerative disorders (Menuet et al., 2011).

USVs are typically recorded in unfamiliar sound-proof chambers for short periods. By recording USVs in the home cage, one can significantly refine studies by taking advantage of prolonged recordings in a familiar environment. Currently, the main challenges are to minimize the effect of USV reflections caused primarily by the cage walls and objects (Hoffmann et al., 2012), improve detection of USVs in noisy recordings (Tachibana et al., 2020) and to relate USVs to individual behavior when animals interact (Vendrig et al., 2019). Hobson et al. (2020) provide an example of recording USVs in socially housed mice in an IVC cage. Their system is not designed to determine the identity of the caller, however the use of multiple microphones to triangulate sounds has been shown to provide accurate identification. A few solutions have been developed, although for use outside of the home cage (Sinelnikov et al., 2015; Heckman et al., 2017; Warren et al., 2018; Sangiamo et al., 2020). USVs can be analyzed in software like Avisoft-SASLab Pro (Avisoft Bioacoustics) and UltraVox XT (Noldus). To date, few studies have combined USVs with tracking data in multi-day, home cage observations (Peters et al., 2017). Recently, neural networks have been designed to detect and classify USVs (Coffey et al., 2019; Ivanenko et al., 2020).

## Measuring and Analyzing Behavior

### Basic Readouts of Video-Tracking

Home-cage monitoring systems enable the automated and multimodal measurements of behavior to occur throughout the day in an entirely experimenter-free manner. Among the many readouts that are available (particularly with home-cage instrumentation), those derived from center-point tracking are perhaps the most dynamic. With x-y coordinates typically sampled at a predetermined rate ranging 5–30 Hz, those that focus on horizontal displacement include distance moved (cm/epoch) and velocity (i.e., mean sample velocity during that epoch). The same datasets are automatically applied to measure various features of horizontal displacement, including acceleration, “meandering” and angular velocity. Measurements of movement (distance per time unit) allow for estimates of “sleep” as described below (Pack et al., 2007; Jankovic et al., 2019; Bass et al., 2020), as well as enable the appreciation of the structure and morphology of active states (Goulding et al., 2008; Hillar et al., 2018). Thus, two groups of rodents with similar total daily horizontal displacements may in fact have very distinct rhythms of rest and activity. Furthermore, measurements of movement derived from changes in pixel intensity of the tracked object (“mobility”) or those of the entire field (“activity”) can also be applied. Changes in mobility may be more sensitive to movements that do not accumulate horizontal displacement (Jankovic et al., 2019).

Position data can also be applied to study the cumulative time spent within (or entries into or out of) one or more predefined zones. In this manner, one can assess other parameters such as feeding or drinking behavior (Robinson et al., 2008). With a combination of lickometers and feeding meters, both the frequency and duration of eating or drinking behavior can be tallied automatically (Jankovic et al., 2019; Bass et al., 2020). With regards to sheltering an opaque shelter can be defined in EthoVision as a hidden zone. Circadian rhythmicity can be explored by calculating hourly values of shelter time (time spent inside the shelter in second). Moreover, we can determine frequency and time spent on top of the shelter, and cage floor movement (time spent moving on the cage floor in seconds), which are characteristic of exploratory and spontaneous locomotor activity (de Visser et al., 2006; Dalm et al., 2009; Manfré et al., 2017). The implementation of a home cage shelter and dynamic alterations of sheltering (using infrared-translucent shelters) also provides a valuable second dimension besides movement when measuring home-cage responses to particular stressors (see the “light spot test” in Section “Measuring Anxiety”).

In a study on Autism Spectrum Disorders (ASD), several behavioral readouts were scored in PhenoTyper by EthoVision XT, including circling behavior, expressed as the frequency of circling, rearing, movement and time spent in walking. These parameters represent hyperactive and repetitive phenotypes, which are behavioral abnormalities observed in ASD in humans (Arroyo-Araujo et al., 2019). Events may be difficult to detect; T-pattern analysis finds “hidden” patterns of behaviors at different time scales (Casarrubea et al., 2018); stereotypies have been detected in the home-cage with this methodology (Bonasera et al., 2008). Beyond movement, home cage instrumentation for measuring food consumption and drinking bouts help to clarify whether hypo- or hyperactivity are associated with mirrored changes in neurovegetative function (Section “Food and Water Consumption”).

Further, as highlighted in Section “Measuring Learning and Cognitive Functions”, a variety of cognitive measurements can be assessed within the home cage, including precise measurements of impulsive/compulsive behaviors as well as aspects of discrimination learning (Remmelink et al., 2016b, 2017). In the discriminative avoidance task, counting the number of shelter entries to the left and right entrance is considered valuable for evaluating cognitive behavior (de Heer et al., 2008).

Various social related tests can be performed in the PhenoTyper. In order to analyze social behavior of animals using the social odor discrimination test, extracting parameters including duration for sniffing and presence of subject’s nose within odor presented zone, latency to first approach, total number of visits to each odor can be useful. In the direct social interaction test, we can quantify behaviors like following/being followed, sniffing and attacking (Harrison et al., 2020). In the Section “Social Behavior in the Home Cage” we address the challenges of automatic assessment of social behavior.

### Measuring Anxiety

Anxiety disorders are the most prevalent psychiatric disorders (Thibaut, 2017) with a worldwide average prevalence around 7.3% and are highly comorbid with other conditions, such as psychiatric disorders (Braga et al., 2013; Wu and Fang, 2014; Koyuncu et al., 2019), substance use disorders (Smith and Book, 2008; Smith and Randall, 2012), dementia (Kwak et al., 2017) and others (Bajor et al., 2015; Yan et al., 2019). Anxiety disorders, categorized in various subtypes (Thibaut, 2017) are often characterized by symptoms such as nervousness, apprehension, difficulty to concentrate, motor tension, and an overall feeling of stress.

**Figure 4:**
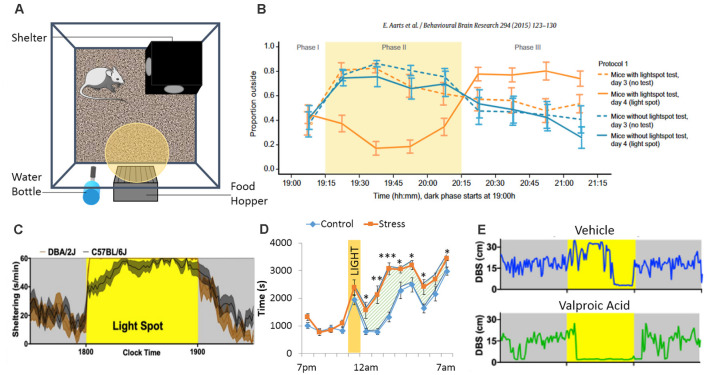
The PhenoTyper boxes are equipped with a shelter, a food hopper, a water bottle, a yellow (or white) LED light and a ceiling mounted camera allowing tracking of the animals **(A)**. During the dark phase, the LED light can be turned ON, shining above the food zone. This creates a conflict between food intake and the subject’s fear of lit environment. In the Light Spot Test **(B)**, animals show reduced time spend outside of the shelter when the light is ON. A study from Jankovic *et al* (2019) identified strains differences regarding sheltering time in response to the light test, while other “standard” tests failed to identify strain differences **(C)**. Another way to assess anxiety-like behavior using the PhenoTyper would be to investigate how animals react to the light (as in the Light Spot Test) as well as their behavior when the light is turned back OFF. Residual avoidance behavior can be observed **(D)** in some cases, like after chronic stress exposure, where mice tend to stay in the shelter even when the light has been turned off, suggesting the presence of more pervasive anxiety-like behavior (the hatched area highlights the residual avoidance period *p < 0.05; **p < 0.01; ***p < 0.001). Finally, the Light Spot test can be performed with pairs of mice tested in the same PhenoTyper (without the shelter to ease tracking). During the light challenge, animals receiving valproic acid prenatally spent more time close to each other (DBS: distance between subjects), compared to animals receiving vehicle **(E)**. Figures are redrawn from Aarts et al. (2015), Bass et al. (2020), Jankovic et al. (2019), and Prevot et al. (2019b).

Clinical (Holzschneider and Mulert, 2011) and preclinical (Lister, 1987; Cryan and Holmes, 2005; VanElzakker et al., 2014) models have been used to study anxiety disorders. Animal models (Cryan and Holmes, 2005) were developed to improve our understanding of the pathophysiological mechanisms underlying anxiety (Bailey and Crawley, 2009; Lezak et al., 2017; Nikolova et al., 2018), and to develop anxiolytic treatment (Prevot et al., 2019a; Biggerstaff et al., 2020; Lorigooini et al., 2020a,b). However, the variability in the assessment of anxiety-like behaviors in animals generated conflicting findings (Ohl, 2005; Steimer, 2011). This variability in results is in part due to the high number of different procedures used between laboratories, species/strain differences and experimenter-biases (Ohl, 2005). O’Leary et al. (2013) demonstrated that anxiety traits of inbred mouse strains are best reflected by species-typical behaviors in each apparatus, suggesting that different tests assess different subtypes of anxiety and are not always reliable. To address this issue, automated, longitudinal approaches in a home-cage setting may help reduce variabilities associated with handling stress and other protocol-related inconsistencies (such as arena size, test duration, etc.). In addition to measures of spontaneous unperturbed home-cage behavior, PhenoTyper cages are also amenable to extract features of conflict-induced behavior that may capture components of anxiety-like behavior. The most popular approach thus far has been the “light spot test” (Aarts et al., 2015; Kyriakou et al., 2018; Jankovic et al., 2019), where a bright light is programmed to be presented within the home-cage (targeted to the drinking and feeding zone) during the dark phase of the light/dark cycle (Figures 4A,B). Rodents being nocturnal animals, avoid lit environments and proceed to hide in the shelter, decreasing drinking and feeding behaviors (Jankovic et al., 2019). Acute injections of diazepam, a drug with acute anxiolytic properties (Tallman et al., 1980), attenuates this light-induced sheltering response and enhances exploration outside of the shelter in spite of the light stimulus. Compared with C57BL/6J mice, one study found that DBA/2J mice display a more robust and rapid light spot-response (Jankovic et al., 2019; Figure 4C), an effect that was not previously noticed using more conventional anxiety-testing (O’Leary et al., 2013). In another example, PhenoTypers were modified to have a sheltered and non-sheltered feeding place, allowing dissociation of motor activity levels and preference to shelter both during novelty and following adaptation to the home-cage environment. Genetic mapping revealed a gene, Adenylyl cyclase 8 (*Adcy8*), for this sheltering feeding behavior that was associated with mood disorders in humans, reflecting its translational value (de Mooij-van Malsen et al., 2009).

Prevot et al. (2019b) showed that animals exposed to chronic stress and non-stressed animals have a similar immediate response to the light challenge, but the former exhibit lasting avoidance behavior when the light switches off, demonstrating a more pervasive and enduring sheltering response (Figure 4D). This behavior, termed residual avoidance, is observed in various models of chronic stress and across various mouse strains, while other behavioral tests like the elevated plus maze or the open field are less consistent between strains or even between experiments (Eltokhi et al., 2020). Residual avoidance was reversed by chronic treatment with the antidepressant imipramine, which has shown efficacy at reversing anxiety in human patients (Hoehn-Saric et al., 1988; McLeod et al., 2000) but not diazepam. This suggests that improvements in residual avoidance may serve as a behavioral biomarker for the long-term adaptive neuroplastic changes that accompany chronic antidepressant intake. The light spot test can also be employed to study stress responses within pairs (or dyads) of mice, where two mice are housed together in the PhenoTyper without shelter (Bass et al., 2020). When faced with the same light spot stimulus, pairs of adult mice prenatally exposed to valproic acid displayed increased inter-mouse proximity compared with control mice (Figure 4E). This effect was not associated with more pervasive changes in proximity or social withdrawal, and no differences in light spot behavior or tone-induced sheltering were seen when mice were studied in isolation (Bass et al., 2020).

Overall, such advances in automated, non-invasive, experimenter-free approaches to assessing anxiety-like behavior within home-cage settings are well poised with respect to rigor and reproducibility. Combined with other measures, such as simultaneous EEG, ECG or pneumoplethysmography, PhenoTyper-based assessments may refine our understanding of behavioral states and could contribute to better understanding of underlying mechanisms involved in anxiety and stress-related outcomes.

### Food and Water Consumption

Assessment of food and water consumption are important biomarkers of general wellbeing, and are also important aspects of spontaneous behavior that may vary with genetic and pharmacological interventions. Traditional methods for assessing food and water consumption in animals relied on fasting of animals followed by short-term measurements of food/fluid intake. These strategies may be associated with altered emotionality related to nutrient deprivation, as well as the novelty-inducing aspects of the test cage. Home cage video observation systems such as the PhenoTyper allow the two approaches to be combined and offer the additional advantage of continuous and handling-free monitoring of behaviors including feeding, drinking and ambulatory activity.

**Figure 5:**
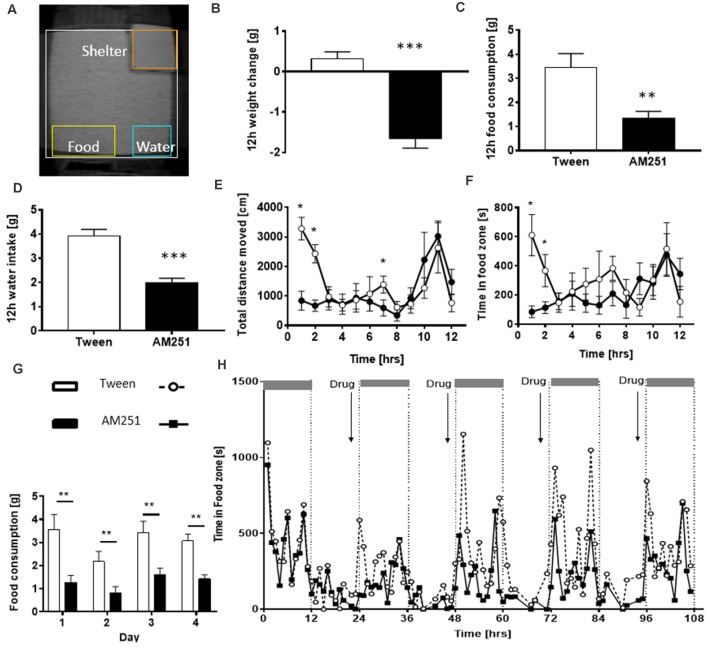
Assessment of food and water consumption in the PhenoTyper. **(A)** Home-cage arena **i** ndicating the location of defined zones of interest including food and water zones adjacent to the food hopper and water bottle. Treatment with AM251 induced a decrease in body weight **(B)**, food intake **(C)** and water intake **(D)**. They also spent less time in the food zone compared with controls **(E)** and displayed lower levels of ambulatory activity **(F)**. Repeated administration of AM251 suppressed food intake **(G)** with home cage observations indicating a reduced time in food zone each night following drug treatment **(H)**. The figure is adapted from Riedel et al. (2009). *P < 0.05; **P < 0.01; ***P < 0.001.

When the PhenoTyper was first developed in the early 2000s, traditional proxies of food and water consumption were reverted to by weighing the hoppers and bottles manually. Yet at the same time, innovative surrogate measures were utilized to detect and estimate food or water intake as a correlate of time spent in pre-designated zones adjacent to the hopper or bottle (Riedel et al., 2009; Robinson et al., 2013). Pharmacological intervention trials exploring the hypophagic/hyperphagic properties of cannabinoid receptor antagonists (AM251, ABD459) or plant cannabinoids like Δ^9^-THCV (Riedel et al., 2009; Goonawardena et al., 2015) involved the measurement of food and water intake in response to either acute or repeated drug administrations and continuous longitudinal recordings (Figure 5). Utilization of EthoVision XT software enabled a direct correlation analysis between food/water consumption measured manually and time spent in and circadian occurrence bouts to the pre-defined food zone/water zone thereby defining these video-based proxies as surrogate measures for food and water consumption. Moreover, they strongly correlated with body weight gain/loss and were sufficient to establish hypophagic/hyperphagic drug properties. Interestingly, the reduction of feeding with AM251 was associated with a reduction in movement. This brings up the issue of which causes which: can reduced feeding be explained by reduced movement or *vice versa*?

**Figure 6:**
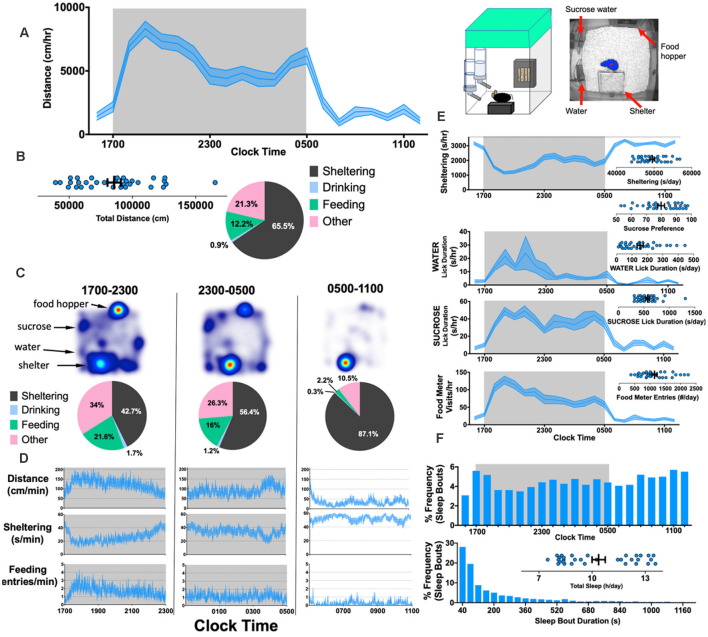
Simultaneously measuring kinematic and neurovegetative function in PhenoTyper home cages. Right: Cartoon showing home-cage configuration with a screen capture from an aerial infrared camera showing mouse body contour (blue) and center point (red). **(A)** Horizontal distances accumulated hourly by 8-week old C57BL/6J mice (*n* = 32, 16 female). **(B)** Total distances moved (per day), and the mean “time budget” calculated across this 21 h recording period. **(C,D)** Heat maps, time budgets and behavioral quantities depicted over 6 h long epochs. “Other” is defined as time spent *not* sheltering, drinking or feeding. **(E)** Average rates of sheltering, licking and feeding measured simultaneously with individual total values plotted in inset. **(F)** Percent frequency of (noninvasively derived) sleep bouts as a function of time of day (Top) and by duration of sleep bout (Bottom), with individual values obtained for total sleep (Inset). The mean + SEM is shown. The figure is adapted from Jankovic et al. (2019).

This work has been followed up by recent studies in a more improved/advanced version of the PhenoTyper containing two separate lickometer waterspouts and a feeding monitor with a beam break device allowing for the automatic recording of water intake and feeding behavior. Similar, to observations with the original system, Krishnan and colleagues (Jankovic et al., 2019) confirmed that drinking and feeding behavior in PhenoTyper home cages are generally synchronized with locomotor activity and either parameter could be sufficient to independently extrapolate circadian rhythms (Figure 6). Results revealed differences in the duration of various activities, with a typical C57BL/6 mouse spending about 10% of the day eating and 1% drinking, although this is different for other genetic backgrounds (Goulding et al., 2008). With two water sources, it is also possible to assess hedonic-like behavior by measuring sucrose preference (Bass et al., 2020). Thus, in summary, HCMS-based assessments of feeding and drinking behavior have numerous advantages, including: (i) removing the need for food/water deprivation; (ii) enabling a cross-sectional design and within-subject analysis allowing for “wash out periods”; and (iii) enabling putative measurements of taste aversion and tolerability, particularly when studying the effects of compounds dissolved in drinking water (Bass et al., 2020). Overall, the HCMS has proven to be a reliable and sensitive test system for assessment of food and water consumption.

### Measuring Learning and Cognitive Functions

The automated home cage offers a valuable opportunity to analyze cognitive performance in a standardized setting, as well as non-invasive parameters including diurnal activity, movement, velocity and acceleration that may co-vary with learning (Robinson et al., 2013; Logan et al., 2018). Studying cognitive abilities and its co-varying factors is important to understand conditions where cognition is impaired. Furthermore, the assessment of “age-related cognitive impairment” is a critical scientific research area in human disease. Recent evidence of cognitive impairment in a mouse model of accelerated aging using the PhenoTyper indicates the translational potential for assessing cognitive function in various models of aging and models designed to mitigate age-related changes in learning and memory (Logan et al., 2019; Parks et al., 2020). In addition, when standard measurements like the Cumulative Learning Index (Logan et al., 2019) are proved to be stable over a period of several years, they provide a composite, reproducible measure. Thus, fundamentally more rigorous and powerful testing paradigms are currently being developed using the PhenoTyper that control for many of the experimental caveats present in earlier studies (Chesler et al., 2002a,b; Sorge et al., 2014), thereby permitting reliable interpretation of interventions that affect learning and memory performance with age.

**Figure 7:**
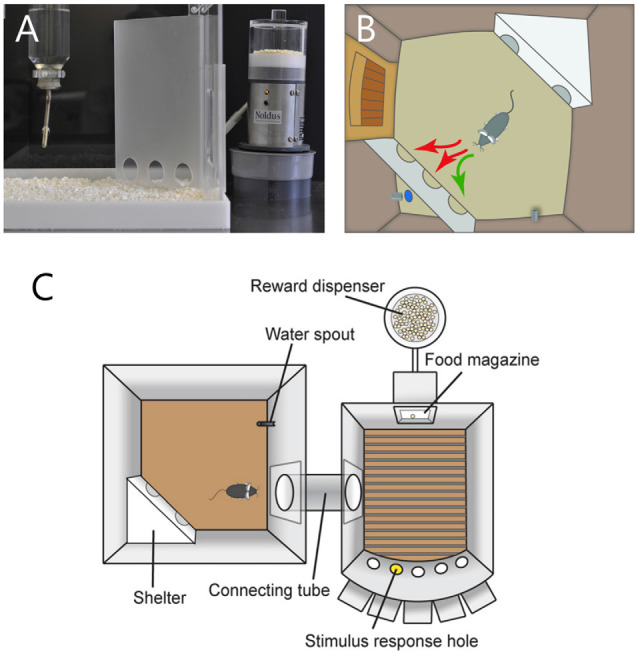
**(A)** The CognitionWall for identifying discrimination learning impairments. The CognitionWall is an opaque Perspex wall with three entrances that is placed in front of a food dispenser inside PhenoTyper. **(B)** In the discrimination learning test, mice are rewarded with a food pellet (blue dot) when they choose to pass through one of the three entrances; in this example, the left-most entrance. In the reversal learning test (not shown), the rewarding entrance is switched to another one, for example the right-most entrance. The scheme is adapted from www.sylics.com. **(C)** Top view of the Combicage. Left, the PhenoTyper home-cage. Right, MedAssociates operant chamber. The test animal can move between the two parts through a custom connection tube. The figure is adapted from Remmelink et al. (2017).

A routine learning paradigm for experimentally testing rodent cognition is operant conditioning, where an association is made between a specific behavior and a positive (rewarding) or negative (punishing) consequence for that behavior. Appetitive operant conditioning is a form of instrumental learning that is traditionally studied in rodents by using an operant conditioning chamber where the animals have to learn to respond with a lever press or nose poke to a stimulus to receive a food or liquid reward (Hånell and Marklund, 2014). Although operant testing provides in-depth insights into cognition, these traditional operant learning paradigms rely on labor-intensive animal handling and commonly require food-restriction protocols to promote the motivation of rodents to learn, which increases stress levels and changes in circadian and task-related activity patterns in rodents (Kant et al., 1988; Hashimoto and Watanabe, 2005; Hut et al., 2011; Guarnieri et al., 2012). Operant learning can simply be introduced in the PhenoTyper by programmed delivery of a reward in the reward zone of the home cage when the animal makes an instrumental response (Remmelink et al., 2015). A standardized test operational in the PhenoTyper is the CognitionWall (Figures 7A,B). Following initial assessment of basal behavior, the CognitionWall is placed in front of the reward dispenser. Subsequently, animals need to learn to earn a reward by passing through one of the holes, while entering through the other two holes does not result in a reward. The difficulty of the task can be varied by adjusting the number of entries required to receive the reward (Remmelink et al., 2016b). This type of discrimination learning likely relies on plasticity in several brain regions and since it is spatially cued this may include the hippocampus. Consistent with the notion that hippocampus-dependent spatial reference memory is one of the earliest impairments in Alzheimer’s disease, a mouse model for early Alzheimer (APP/PS1-transgenice mice) showed a significantly reduced capacity for discrimination learning in this task (Remmelink et al., 2016b).

The CognitionWall can subsequently be used to test rodents for their capacity for reversal learning, a prime aspect of cognitive flexibility (Klanker et al., 2013), by simply switching the reward delivery to one of the other two entry holes. Similarly to humans who require more time to change strategy than to learn the initial strategy (Dias et al., 1997; Tsuchida et al., 2010), mice require more entries to reach the 80% criterion for reversal learning in the CognitionWall than necessary for discrimination learning, although they can achieve this criterion within 3 days (Remmelink et al., 2016b). In addition, mice make more perseverative errors compared with neutral errors during reversal learning (Remmelink et al., 2016b), similarly as is observed in humans undergoing reversal learning (den Ouden et al., 2013). In humans this type of flexible stimulus-reinforcement learning is known to rely on the orbitofrontal cortex (OFC; Hornak et al., 2004; Tsuchida et al., 2010). OFC lesions in mice significantly impair reversal learning in the CognitionWall task, while leaving discrimination learning and general activity intact (Remmelink et al., 2016b). These findings therefore validate this reversal-learning task in the automated home-cage for translation to the human situation. Mouse models of accelerating aging (SOD1-knockout mice) show a selective deficit in reversal learning (Logan et al., 2018), suggesting that aging may primarily cause a problem in behavioral flexibility. An interesting observation was made in reversal learning for mice in which fatigue is induced by pelvic irradiation (Wolff et al., 2020): fatigued mice showed reduced performance in the task, but not because of a learning deficit but because they engaged the task at a slower pace, illustrating the value of the PhenoTyper being able to distinguish between the two.

The PhenoTyper has also been successfully used to study impulsivity and attention in rodents (Remmelink et al., 2017; Bruinsma et al., 2019). Deficits in attention and impulse control are hallmarks of psychiatric disorders such as schizophrenia and attention-deficit hyperactivity disorder (ADHD; Castellanos and Tannock, 2002; Luck and Gold, 2008). A standard behavioral paradigm that is used to test motor impulsivity and visuospatial attention in rodents is the 5-choice serial reaction time task (5-CSRTT), in which animals have to correctly identify *via* a nose poke which of the five holes has been briefly illuminated to receive a palatable reward (Robbins, 2002). However, conventional 5-CSRTT paradigms rely on food-restriction and human intervention, and typically take several weeks for animals to accomplish. By linking the home-cage *via* a tunnel to a 5-CSRTT chamber (Figure 7C), both mice (Remmelink et al., 2017) and rats (Bruinsma et al., 2019) were allowed to execute this task at their own pace, which led to significant reduction in time to complete the task to at most one week. The accuracy in completing the 5-CSRTT is significantly reduced upon the injection of scopolamine, a drug that blocks muscarinic acetylcholine receptor and that is known to impair attentional control, providing pharmacological validation of the task (Remmelink et al., 2017; Bruinsma et al., 2019). The concept of the home-cage being coupled with a separate test chamber could be applied to develop other types of home-cage operated tests (e.g., Schaefers and Winter, 2011), including those that require the test to be spatially separated from the home cage, like in the contextual fear conditioning.

**Figure 8:**
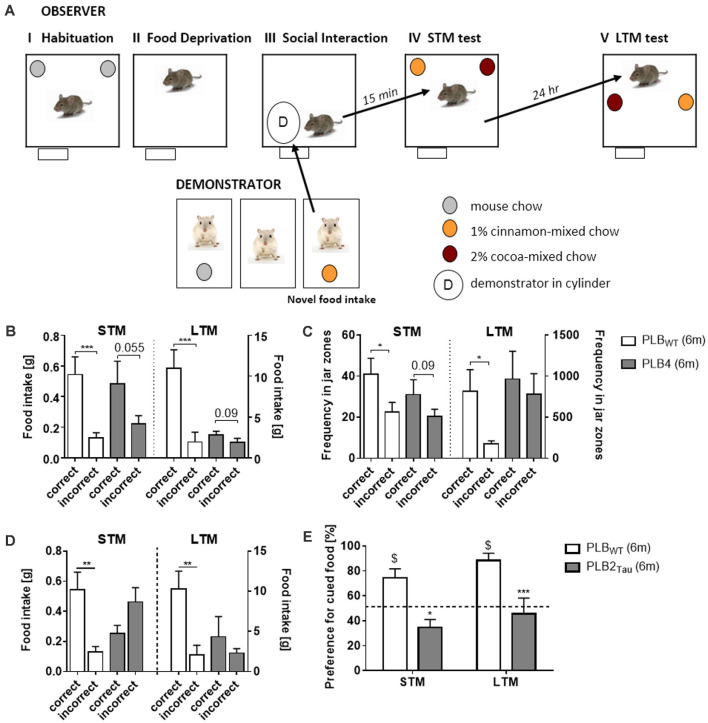
Assessment of semantic-like memory in a home-cage environment *via* a social transmission of food preference (STFP) task. **(A)** Outline of a novel semi-automated STFP task developed in the PhenoTyper. The task consists of various phases using “observer” and “demonstrator” animals. Observer animals are initially habituated to the PhenoTyper whilst demonstrator mice are single housed, both animals are habituated to food jars containing mouse chow. Prior to the test all mice are subjected to overnight food restriction after which the demonstrator animals are given a flavored mouse chow (cocoa or cinnamon). The observer animal is subsequently exposed to the demonstrator animal *via* a cylinder positioned within the PhenoTyper and interaction between the two animals initiated. Social interaction for cued food was followed by either a short (15 min—STM) or long delay (24 h—LTM) prior to the mice being tested for recall *via* the presentation of jars containing correct and incorrect food. The amount of food consumed, and time spent in the zones associated with each jar were recorded with intact semantic memory represented by a preference for the cued food they were exposed to *via* the demonstrator. Analysis of correct food eaten, i.e., food matching the flavor of the demonstrator **(B)**, and time spent in food jar zones **(C)** revealed that 6 month old PLB4 mice (mice with mild overexpression of human BACE1 involved in neurodegeneration) displayed impaired memory for the cued food in both STM and LTM tests, with only PLB_WT_ mice (i.e., mice from PLB crossings that do not carry transgenes) displaying intact memory for the cued food. Impairments in memory for the cued food were also observed with PLB2_Tau_ (i.e., knock-in mice which express a single copy of FTD human Tau) with mice consuming less of the correct food **(D)** and in contrast to age matched PLB_WT_ mice they demonstrated no preference for the cued food in either STM or LTM tasks **(E)**. The figure is adapted from Plucińska et al. (2014) and Koss et al. (2016). *P < 0.05; **P < 0.01; ***P < 0.001, for group comparisons. ^$^*P* < 0.05 significance vs. chance (50%).

An early clinical marker for aging and for Alzheimer’s disease is a deficit in olfactory recognition (Bahar-Fuchs et al., 2011). As a test for olfactory learning, Social Transmission of Food Preference (STFP) was developed and semi-automated in the PhenoTyper (Plucińska et al., 2014; Koss et al., 2016) based on the original protocol of Galef and Wigmore (1983). The STFP test in mice measures retrieval of semantic-like memory for olfactory information acquired *via* social interaction. In this semantic memory task, a food preference of an “*Observer*” mouse is induced *via* social interaction with a “*Demonstrator*” mouse previously exposed to distinctly flavored food and the socially acquired olfactory memory is subsequently assessed in a food preference test in the PhenoTyper (Figure 8A). Assessment of STFP in mouse models of Alzheimer’s disease (PLB4 mice; Plucińska et al., 2014; Figures 8B,C) and frontotemporal dementia (PLB2_Tau_; Koss et al., 2016; Figures 8D,E) confirmed impairments in STFP indicated both by food preference and the automatic measurement of time spent in the relevant food associated areas in the PhenoTyper (Figure 8B). These results prove the utility of the PhenoTyper in the assessment of semantic-like memory.

### Social Behavior in the Home Cage

The bulk of home cage-based assessments of cognitive and emotional behavior in rodents have been conducted with socially isolated subjects. This approach removes temporally dynamic sources of variability that would be expected in group-housed settings under free social exploration conditions. Further, it is well positioned for within subject correlations (e.g., is feeding time generally proportional to licking?) or comparisons across subjects designed to highlight individual differences in behavior. In contrast to this traditional approach, more recent studies have indeed conducted prolonged home-cage recordings in social groups of subjects. The vast majority of these approaches have primarily been interested in the activity patterns of the group (overall; Ahloy-Dallaire et al., 2019; Pernold et al., 2019). Discriminating between mice within a group has more recently been made possible through the use of radiofrequency identification chips (RFID). When combined with video tracking, this technology provides objective measurements of horizontal distances, feeding and licking behavior for individual mice within a group, as well as measures of social behavior (Alexandrov et al., 2015; Peleh et al., 2019). Using this approach, Kas and colleagues demonstrated that BTBR mice engaged in far fewer social behaviors (like sniffing, approaching or interacting with each other) compared with C57BL/6J mice (Peleh et al., 2020). Using depth camera image analysis, it is possible to overcome the limitations imposed by 2D video tracking (occlusions, vertical movement) to define more complex social behaviors (de Chaumont et al., 2019; Hong et al., 2015), although this approach relies on machine learning and probably requires independent training sets for different rodent inbred strains (see “Today and the Future” Section).

In studies employing PhenoTyper home cages, a variety of protocols have been employed. One study conducted brief assessments of juvenile play in mice at 21 days of age, and demonstrated that the phase of testing (day vs. night) exerted variable effects on manually scored social interactions (Yang et al., 2007). Krashes’ group directly assessed the prioritization of feeding and social behaviors within PhenoTyper cages. With optogenetic stimulation provided through ceiling holes and programmed *via* EthoVision XT, patterns and sequences of feeding and social measures were assessed manually, including aggressive and mating behaviors (Burnett et al., 2019). By defining a social interaction zone around a mouse concealed in a wire cylinder, two studies have demonstrated that social exploration time can be objectively measured within a natural setting (Plucińska et al., 2014; Koss et al., 2016). And finally, by applying the social interaction module within EthoVision XT, one group demonstrated the feasibility of an entirely noninvasive videotracking-based approach to study home-cage social behavior (Bass et al., 2020). In this particular example, adult mice that were exposed prenatally to valproic acid were studied as pairs (“dyads”). In comparison with control mice, valproic acid exposure produced a deficit in inter-mouse proximity during a very transient circadian period (around the time of the dark-light transition). Inter-mouse proximity was also interrogated during the light spot test (see "Measuring Anxiety" Section), providing additional measures of sociability changes during conflict stimuli. The PhenoTyper and similar cages could also be used in social defeat stress testing (e.g., Golden et al., 2011). Although we are not aware of publications featuring such protocols in the PhenoTyper, one could divide the cage into two halves and ensure that both the intruder and the aggressor have access to a water/food source. The consequences of social defeat could be evaluated in the home-cage too.

Whether RFID technology is applied or not, studies thus far have focused on groups of condition-matched subjects (e.g., groups/pairs of BTBR mice vs. groups/pairs of C57BL/6 mice). This iteration probably most drastically reveals pharmacological or genetically mediated phenotypic differences. In the future, we anticipate advances in automated analyses of mixed populations of subjects, particularly within natural home-cage settings such as the classical visible burrow system (McEwen et al., 2015). This will be essential to improve the translational potential of preclinical studies in neuropsychiatric disorders that impact social function.

### Home Cage Testing for Unraveling the Genetics of Behavior

The PhenoTyper provides the possibility to investigate within and between strain variation in genetically mutant animal models. One example is a study that monitored spontaneous home-cage behavior of 11 inbred strains of mice (129S1/SvImJ, A/J, C3H/HeJ, C57BL/6J, BALB/cJ, DBA/2J, NOD/LtJ, FVB/NJ, WSB/EiJ, PWK/PhJ and CAST/EiJ) in the PhenoTyper and assessed between strain variation to find the influence of genetic factors on activity-related phenotypes, yielding 115 behavioral parameters of which 105 revealed highly significant strain differences. Especially for sheltering behavior, large genetic effect sizes were observed. For instance, it was found that 129S1/Sv, A/J and C3H/HeJ strains did not climb on top of the shelter and their motor function was also not impaired (Loos et al., 2014). This study demonstrates that home-cage behavioral analysis is able to detect genetic/strain effects that cannot be easily studied using conventional behavioral tests. Another study of four inbred mouse strains (C57BL/6, DBA/2, C3H and 129S2/Sv) provided the evidence that circadian rhythmicity, novelty-induced activity and the time-course of specific behavioral elements are different between strains. For instance, activity decreased faster over time in C57BL/6 and DBA/2 mice compared to C3H and 129S2/Sv mice. A principal component analysis revealed that there were two major factors within locomotor activity, namely “level of activity” and “velocity/stops”, which distinguished the different strains (de Visser et al., 2006). Furthermore, a study of eight different isogenic strains of mice observed significant differences in phenotypic robustness (Loos et al., 2015). Scoring the behavior of animals in the PhenoTyper enabled researchers to assess the activity of hybrid animals and compare them with their parental strains. CB6F1/6J is a hybrid mouse model which comes from breeding two inbred mice (C57BL/6J and BALB/cJ). This animal showed similar phenotypes to both parents. However, their horizontal activity in the home cage closely resembled that of C57BL/6J mice (Tang et al., 2002). In addition to utilizing the PhenoTyper system to assess strain differences, there has been an increase in studies assessing the behavior of genetic mouse models for various diseases including Alzheimer’s disease (PLB4, PLB2APP; Plucińska et al., 2014; Plucińska et al., 2018), frontotemporal dementia (PLB2-_Tau_; Koss et al., 2016) and Rett Syndrome (Mecp2; Robinson et al., 2013). Home-cage analysis within the PhenoTyper facilitated the identification of behavioral phenotypes including alterations in circadian and ambulatory activity which are core symptoms of these diseases.

While mice are traditionally used to define the contribution of specific genes to behavior, rats are on the comeback. Rats offer the advantage over mice that their behavioral repertoire is more elaborate (Homberg et al., 2017). Serotonin transporter knockout (SERT^−/−^) rats tested in the PhenoTyper presented with increased anxiety and cocaine-induced locomotor activity compared to wild-type controls. Furthermore, the knockout rats explored the PhenoTyper cage by ceasing movement and scanning their environment. Interestingly, crossing the knockout and wild-type counterparts having a Wistar background with Brown Norway rats altered these behavioral manifestations and led to the identification of SERT^−/−^-specific quantitative trait loci (QTLs) for parameters related to the behavioral manifestations (Homberg et al., 2010). More recently, a dopamine transporter knockout rat model has been generated, displaying pronounced hyperactivity and cognitive dysfunction (Leo et al., 2018). The hyperactivity, however, appears to be age— and context-dependent. A detailed assessment of the behavior in the PhenoTyper is expected to leverage a detailed view on the activity-related alterations in this rat model.

### Analysis Scripts and Meta-Analysis of Large Datasets

The wealth of data obtained during hours or days of home-cage monitoring provides opportunities for an even more diverse set of data analyses techniques, and hence greater heterogeneity in studies reporting these outcomes. Multi-day experiments in PhenoTyper cages have spurred the development of additional analyses scripts that specifically analyze aspects of behavior that are not captured in typical studies of a few hours, including circadian rhythms and sheltering behavior. Trial control functions make it possible to create complex customized testing protocols, and these custom protocols require dedicated analysis scripts in order to generate relevant outcome measures. At present, it is customary for developers of testing protocols and analyses scripts to describe these in detail in scientific publications. However, converting a textual description from a materials and method section into an analysis script is not an unambiguous process, and is bound to lead to differences in outcomes. One way forward could be sharing of scripts through repositories such as GitHub. For users that are not into scripting, commercial solutions are available such as the fee-for-service platform AHCODA that can be used to process raw data to standardized outcome measures frequently reported in literature.

With the increasing availability and use of standardized home-cages over the last decades, the first systematic comparative studies have been published that provided new insights that could not have been obtained using single datasets. An example of a within-laboratory systematic comparison was a study into the within-strain variability in spontaneous behaviors of eight common inbred strains of mice (Loos et al., 2015). By collectively analyzing dozens of cohorts of these strains, it became apparent that some strains display less within-strains variability and are more homogeneous, such as for instance C57BL/6J mice in comparison with other strains such as the DBA/2J strain. Although this is an example of a systematic comparison that required the level of standardization that is achieved with home cages across a time span of multiple years, the data logistics surrounding this systematic analysis was relatively straightforward because all data was acquired within the same laboratory. Comparing data from different laboratories is considerably more challenging and requires identical set-ups, standardized protocols and a central data repository (see Robinson et al., 2018).

To offer web-based data mining and analysis tools for the wealth of quantitative data gathered by individual laboratories and international research consortia using automated home-cages, at the resolution of individual mice, the “AHCODA-DB” data repository was established (Koopmans et al., 2017). This data repository has accessibility at the resolution of individual mice and is open access, which enhances transparency (i.e., enables in-depth post-publication peer review to enhance reproducible science), and allows systematic meta-analyses to generate and test new hypotheses. For example, one can compare datasets of spontaneous behavior of mutant mouse lines measured during multiple days. Mutants that are known to be very similar may line up close together potentially as consequence of the shared underlying molecular and cellular mechanisms that affect behavior. This resource and related tools should allow individual scientists and consortia conducting experiments with common inbred strains and/or mutant lines in PhenoTyper home-cages to systematically compare their data across time, laboratories and experiments.

## An Example of Potential Applications: Neurotoxicological Assays

Current approaches to screening for neurotoxicological effects of environmental chemicals and safety and tolerability of drugs are costly and labor-intensive. They rely on the use of animal models since it is difficult to determine the levels of neurotoxin exposures in humans. Researchers are therefore able to control windows of exposure, dosing, and confounds such as age or mixture effects. However, basic neurotoxicology research often focuses narrowly on a few outcomes utilizing traditional behavioral assays and may miss broader, more translationally relevant effects. For regulatory purposes, developmental neurotoxicology studies for a single environmental exposure can cost several million dollars. Automated home-cage monitoring may provide a novel solution to this problem by producing more meaningful behavioral endpoints and a more high-throughput, scalable approach. Circadian activity, locomotion, and social behavior are important indicators of brain structure and function, so changes in these basic processes can help determine neurotoxicological and pharmacological effects (Graham et al., 2018). Additionally, differing laboratory environments affect rodent behavior (Arroyo-Araujo et al., 2019), so assessing neurotoxicological assays in HCMS during the light-dark cycle could help to minimize effects from external factors.

Despite increasing concerns about environmental influences on the nervous system and cognitive function (Liu and Lewis, 2014), limited research has been done to study neurotoxicity in rodents through behavioral assays using automated home-cage environments. Automated HCMS used in this line of research include TSE IntelliCage (Endo et al., 2012; Aung et al., 2016) and the PhenoTyper (Salvetti et al., 2012; Shiotani et al., 2017). For example, Shiotani et al. (2017) recorded mice for 24 h in the PhenoTyper and measured time and frequency of zone entries, mobility and posture to show that prenatal exposure of domoic acid altered the subjects’ circadian activity patterns, with increased bouts of resting in the dark phase and higher activity levels in the light phase. Through a combination of prolonged recordings and cage instrumentation, including lickometers and feeding meters, these systems also allow researchers to look at the psychopharmacological effects of precisely timed drug and toxin exposures across multiple time scales. Salvetti et al. (2012) analyzed the performance of mice exposed to Quantum Dots in Novel Object Recognition tests conducted in the PhenoTypers. Repeated assessment revealed a decrease in NOR performance (that is, time spent exploring the novel object relative to the total exploration time) only at 3 weeks after injection, again demonstrating the importance of repeated measurements in familiar environments. Despite these benefits, a limitation of HCMS is that they currently lack a uniform recording system. Video tracking software such as EthoVision XT paired with PhenoTyper may produce different behavioral results compared to RFID-sensing that is used by TSE IntelliCage. According to a comparison study by Robinson and Riedel (2014), PhenoTyper has higher spatial resolution than these other systems and therefore may be better for determining drug sensitivity in terms of activity, exploration and fine motor behavior. However, RFID-based systems are suitable to test the effect of chemicals on social behavior where individual identification is required, for example when many individuals compete for access to a resource (Endo et al., 2012). HCMS also cannot test many different drug doses efficiently, requiring preliminary drug testing (Shiotani et al., 2017). One solution to this is the chronic administration of drugs which would allow for increases in drug dosage over time. Oral administration of drugs or toxins through a lickometer could measure how a drug affects behavior in the home cage at varied rates throughout each day and would allow for a between-subjects comparison (Bass et al., 2020). To test prenatal exposure to chemicals, providing pregnant females with toxic drinking water or implanting osmotic pumps before pregnancy could be viable options before moving the pups into an automated home-cage system (Aung et al., 2016; Hawkey et al., 2020). Applying automated home-cage technology to the field of neurotoxicology and neuropharmacology could improve the efficiency and validity of behavioral studies in determining drug exposure effects on brain development and function.

## Today and The Future

Home-cage monitoring systems have proven to reshape the way we view the study of behavior of rodents by helping to standardize behavioral testing in labs across the world (Arroyo-Araujo et al., 2019). First, we have shown that HCMS have the potential to improve reliability and reproducibility of scientific research through automation of data acquisition, based on continuing advances in technology development. Second, HCMS allow streamlining of behavioral testing in various domains, including locomotion, social interaction, food and water consumption, anxiety-like behaviors and cognitive performance. Home-cage studies may provide additional value to the results of standard tests, by: (1) clarifying the effects of cage habituation; (2) tracking ultradian, circadian and infradian oscillations; (3) richly capturing the entirety of spontaneous and provoked behavior at night without the experimenter needing to be present; (4) providing free access to operant training protocols that can be completed over the entirety of the night time period; (5) providing a holistic viewpoint of behavioral manipulation (e.g., the behavior can be measured before, during, and after presentation of a stimulus); (6) permitting a precise exploration of factors that may introduce variability (e.g., testing with different cage sizes, or enrichment factors); and finally (7) allowing multiple opportunities to standardize behavioral measurements.

When looking at the future of behavioral testing, we should first tackle the current limitations, which include, among others, the level of human interference, the use of specific subjects, and the metrics used in the models. HCMS do not completely eliminate human interference at least for tasks that need to be performed by researchers and technicians, like cage changes, which still represents a potential confounding effect. In addition, it remains the researcher’s responsibility to determine whether automated approaches are the best suited to answer their scientific questions.

One of the most glaring omissions from the vast majority of behavioral studies, including many studies currently using HCMS (Aarts et al., 2015; Kyriakou et al., 2018), is the inclusion of female test subjects. Females are often excluded from studies because of complications (real or perceived) that arise from monitoring the estrous cycle. Despite mounting evidence that including female subjects does not increase the variability of the data, but rather strengthens the conclusions that can be drawn from the experiments, many, if not most, articles still rely on data collected from only male subjects (Beery and Zucker, 2011; Becker et al., 2016; Beery, 2018). HCMS can help alleviate some of those obstacles by monitoring female subjects continuously throughout their cycle. Furthermore, continuous monitoring allows for direct comparisons of male and female behavior outside of the short behavioral intervals captured in most behavioral tests (e.g., activity patterns throughout the day, feeding and climbing behaviors, etc.).

Animal models allow for unique insights into the potential underlying biology of many conditions that afflict humans. Behavioral protocols have been developed over the years to parameterize such qualities as anxiety-like (Pollard and Howard, 1979; Pellow et al., 1985), depression-like (Porsolt et al., 1978; Steru et al., 1985), even models of certain autism-like behavior patterns in rodent models (reviewed by Kazdoba et al., 2016; Nicolini and Fahnestock, 2018). Many of these metrics do not incorporate discrete animal behaviors, but instead focus on generalized behavior patterns (such as measuring “social contact” as a summation of various different interaction behaviors) or using simple locomotor measurements including location, distance and velocity of movement (Spink et al., 2001; Lorbach et al., 2018). While insightful, these measurements impoverish the broad range of behaviors an animal may demonstrate during even very simple behavioral tests. The desire to anthropomorphize animal responses to behavioral tasks can also occlude progress towards understanding behavioral phenomena in species-specific terms. Experimental results, therefore, must rely on the ethologically relevant behavioral repertoire model organisms express. To counter this, new methods of analyzing behavior have been proposed. For individually housed animals, applications exist that can infer more high-level behaviors like grooming, rearing, sniffing, digging etc. (Jhuang et al., 2010; van Dam et al., 2013). Until a few years ago, these measurements were done by classifying carefully designed features using Support Vector Machines or Hidden Markov Models followed by post-processing, resulting in fast and robust solutions. In recent years, breaking actions into behavioral “syllables” has aided in machine learning-based prediction of pharmacological treatments based on behaviors in an open field. This suggests that behavioral readouts may indicate biological interventions at a level more sensitive than detectable even by the human eye (Wiltschko et al., 2020). Recent advances in deep learning for image processing have boosted the field to new heights, especially with the publication of DeepLabCut (Mathis et al., 2018), SLEAP (Pereira et al., 2019, 2020) and DANNCE (Dunn et al., 2021) for the estimation of body points. Follow-up modules B-Soid (Hsu and Yttri, 2019) and SimBA (Nilsson et al., 2020) are now available to train behavior classifiers on body-part pose data. This will also help in shifting focus towards analyzing discrete animal behaviors with distinct relationships to biological variables of interest. The next advances will come parallel to the large amount of recent AI research related to automated agents (e.g., self-driving cars) that need to infer meaningful behaviors of other agents (human or robot) from continuous time-series data. The work by Berman et al. (2014), the Datta lab (Wiltschko et al., 2015; Johnson et al., 2016) and Graving and Couzin (2020) are early examples that take a data-driven approach to unravel behavioral structure or “grammar” in data streams and relate so-called behavior “syllables” or words to behaviors relevant to animal behavior researchers. The availability of open deep learning frameworks, open source libraries and open challenge datasets in the animal domain will help to advance progress. While earlier effort aimed to assess single-animal open field behaviors (Hsu and Yttri, 2019) and social behaviors (Nilsson et al., 2020), there are many potential applications of these tools, including defensive behavior measurement (Storchi et al., 2020), reaching movements (Parmiani et al., 2019), turning behavior (Mundorf et al., 2020). Multi-animal expansions of these tools will allow for more sophisticated behavior analysis between more than one individual, allowing for monitoring group-wide dynamics in various social patterns such as fighting, mating or parenting.

**Figure 9:**
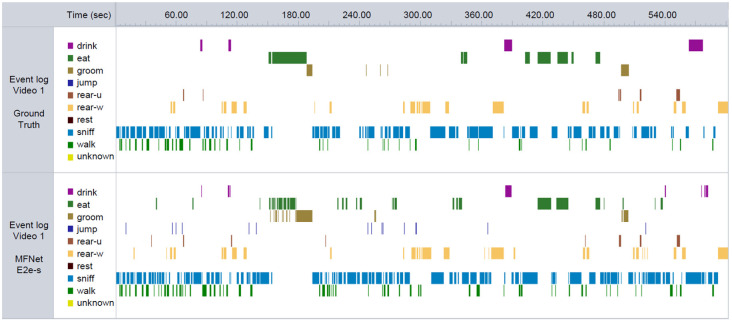
Time plot of behavioral events coded by human observer (Ground truth; top) and scored by the Deep learning annotation system. Note the striking agreement between ground truth and automated annotations. The figure is redrawn from van Dam et al. (2020).

Deep learning algorithms are very good at finding useful cues for what they need to recognize, but such cues may only be visible in specific contexts. This is very difficult to avoid and that makes deep learning models less robust compared to the dedicated feature classifiers, at least for behaviors that are more complex than body points and pose. For the detection of nine behavior categories classes (*groom*, *rear*, *walk* etc.), van Dam et al. (2020) reported an increase in within-experimental-setup performance (see an example in Figure 9) at the cost of a decreased across-setup performance. Currently, all deep learning models for rodent behavior need dedicated training or fine-tuning on the dataset that they are deployed on. HCMS may help as they provide a relatively constant environment. Yet, other types of variances remain, for instance rodent strain, age and sex, and most importantly experimental manipulation that causes animals to behave differently, that is, both the form of the behavior (e.g., intensity and direction) and its temporal structure may vary. Although that may represent a potential limitation, we are confident that methods based on neural networks are the way to go for obtaining more animal-centered measures of behavior. We envision that the sensor technologies, test paradigms and digital behavioral markers explored and validated in HCMS may eventually find their way into the vivarium, thus contributing to the three Rs (Russell and Burch, 1959) and improving scientific validity and reproducibility (Würbel, 2017) of preclinical laboratory studies.

## Author Contributions

All authors listed have made a substantial, direct and intellectual contribution to the work, and approved it for publication. All authors contributed to the article and approved the submitted version.

## Conflict of Interest

BB and QP were employed by F. Hoffman-La Roche Ltd. BK and ML are employed by Synaptologics BV. ED, FG, LN, RFR, and RT are employed by Noldus Information Technology BV. AS participates in a holding that owns shares of Synaptologics BV. The remaining authors declare that the research was conducted in the absence of any commercial or financial relationships that could be construed as a potential conflict of interest.

## Publisher’s Note

All claims expressed in this article are solely those of the authors and do not necessarily represent those of their affiliated organizations, or those of the publisher, the editors and the reviewers. Any product that may be evaluated in this article, or claim that may be made by its manufacturer, is not guaranteed or endorsed by the publisher.

## Footnotes

^1^ www.na3rsc.org
